# Functional CVIDs phenotype clusters identified by the integration of immune parameters after BNT162b2 boosters

**DOI:** 10.3389/fimmu.2023.1194225

**Published:** 2023-05-25

**Authors:** Eva Piano Mortari, Federica Pulvirenti, Valentina Marcellini, Sara Terreri, Ane Fernandez Salinas, Simona Ferrari, Giulia Di Napoli, Daniele Guadagnolo, Eleonora Sculco, Christian Albano, Marika Guercio, Stefano Di Cecca, Cinzia Milito, Giulia Garzi, Anna Maria Pesce, Livia Bonanni, Matilde Sinibaldi, Veronica Bordoni, Serena Di Cecilia, Silvia Accordini, Concetta Castilletti, Chiara Agrati, Concetta Quintarelli, Salvatore Zaffina, Franco Locatelli, Rita Carsetti, Isabella Quinti

**Affiliations:** ^1^ B Cell Unit, Immunology Research Area, Bambino Gesù Children’s Hospital, IRCCS, Rome, Italy; ^2^ Department of Molecular Medicine, Sapienza University of Rome, Rome, Italy; ^3^ Reference Centre for Primary Immune Deficiencies, Azienda Ospedaliera Universitaria Policlinico Umberto I, Rome, Italy; ^4^ Research Biobank, Bambino Gesù Children’s Hospital, IRCCS, Rome, Italy; ^5^ Medical Genetics Unit, IRCCS Azienda Ospedaliero-Universitaria di Bologna, Bologna, Italy; ^6^ Department of Experimental Medicine, Policlinico Umberto I Hospital, Sapienza University of Rome, Rome, Italy; ^7^ Department of Onco-Haematology, and Cell and Gene Therapy, Bambino Gesù Children’s Hospital, IRCCS, Rome, Italy; ^8^ FlowJo, BD Life-Sciences-Biosciences, Ashland, OR, United States; ^9^ Department of Infectious, Tropical Diseases and Microbiology, IRCCS Sacro Cuore Don Calabria Hospital, Negrar di Valpolicella, Verona, Italy; ^10^ Occupational Medicine/Health Technology Assessment and Safety Research Unit, Clinical-Technological Innovations Research Area, Bambino Gesù Children’s Hospital, IRCCS, Rome, Italy; ^11^ Department of Life Sciences and Public Health, Catholic University of the Sacred Heart, Rome, Italy

**Keywords:** CVIDs, antibodies, memory B cells, SARS-CoV-2, COVID-19, mRNA vaccine, booster dose, BNT162b2

## Abstract

**Introduction:**

Assessing the response to vaccinations is one of the diagnostic criteria for Common Variable Immune Deficiencies (CVIDs). Vaccination against SARS-CoV-2 offered the unique opportunity to analyze the immune response to a novel antigen. We identify four CVIDs phenotype clusters by the integration of immune parameters after BTN162b2 boosters.

**Methods:**

We performed a longitudinal study on 47 CVIDs patients who received the 3rd and 4th vaccine dose of the BNT162b2 vaccine measuring the generation of immunological memory. We analyzed specific and neutralizing antibodies, spike-specific memory B cells, and functional T cells.

**Results:**

We found that, depending on the readout of vaccine efficacy, the frequency of responders changes. Although 63.8% of the patients have specific antibodies in the serum, only 30% have high-affinity specific memory B cells and generate recall responses.

**Discussion:**

Thanks to the integration of our data, we identified four functional groups of CVIDs patients with different B cell phenotypes, T cell functions, and clinical diseases. The presence of antibodies alone is not sufficient to demonstrate the establishment of immune memory and the measurement of the in-vivo response to vaccination distinguishes patients with different immunological defects and clinical diseases.

## 1 Introduction

Infection prophylaxis by vaccine administration with inactivated or non-viable vaccines is a current practice in patients with Inborn Errors of Immunity (IEIs) (1, 2). The impaired response to immunization is also included in the diagnostic criteria of many IEIs such as Common Variable Immune Deficiencies (CVIDs) (1, 3, 4), a complex and heterogeneous group of IEIs characterized by hypogammaglobulinemia and impaired antibody production (1, 2). Due to the heterogeneity of CVIDs, humoral immune responses to immunization range from partial to absent antibody production and the grade of impairment correlates with prognosis (5, 6). The antibody defect is treated by immunoglobulin replacement therapy (IgRT) containing a wide spectrum of antibodies (Abs), including those directed to vaccine antigens, with the aim of reducing the number and severity of infections (7). Diagnostic evaluations of humoral responses to vaccines may not be informative in patients with IgRT (8). To overcome this limitation, the analysis of antibody responses to neoantigens has been proposed (9, 10), but rarely used in clinical practice.

Over the last two years, the SARS-CoV-2 pandemic has led to the rapid development of effective vaccines against the viral spike protein, an antigen that humans have never encountered before. Vaccination against this novel antigen has offered the opportunity to study the response to a primary immunization in wide cohorts of patients with IEIs (11, 12), including CVIDs (12–24).

Published data indicate that after two vaccine doses, 60% of CVIDs patients are able to seroconvert (11) with a lower magnitude of antibody response and a reduced virus-neutralizing function compared to control (13–15, 17, 20).

We have no information, however, on whether the presence of serum Abs reflects the establishment of immune memory able to be re-activated by booster vaccination or infection. Vaccine-induced Abs and memory B cells (MBCs) have divergent kinetics in healthy subjects: whereas antibody titers are high early after immunization and rapidly decline, MBCs persist and increase in time (25). In case of breakthrough infection or administration of booster vaccine doses, MBCs rapidly react, increasing in numbers and producing Abs (25). Similarly, memory T cells frequencies and absolute numbers do not decline over time (26).

MBCs include IgM and switched MBCs. IgM MBCs are generated by a T- and germinal center (GC)-independent mechanism, carry few somatic mutations (27–33) and serve as first-line protection against infection (34). Switched MBCs are generated in the GC with the indispensable help of T cells expressing the CD40 ligand (33, 35). MBCs accumulate somatic mutations in the GC and are selected for their increased affinity to the stimulating antigen (36).

In this longitudinal study carried out in CVIDs patients naïve to SARS-CoV-2 infection who completed the primary cycle with the BNT162b2 mRNA vaccine followed by one (3^rd^ dose) or two (4^th^ dose) booster doses, we combine the data on the production of specific Abs and MBCs. We have exploited the extraordinary condition of studying the response to an antigen never encountered before, in a period when immunoglobulins, administered as a substitution therapy, did not contain anti-SARS-CoV-2 IgG as yet.

We demonstrate that serum antibody measurement is not sufficient to predict the establishment of immune memory in CVIDs patients. We also show that the performance of the immune system in the response to vaccination *in vivo* may be used to distinguish patients with different mechanisms at the base of their immune defect and different clinical diseases.

## 2 Materials and methods

### 2.1 Study design and patients

This longitudinal study was carried out on 47 adults with CVIDs diagnosed according to the ESID criteria (37) and regularly followed by the Care Center for adults with IEIs in Rome, Italy. As healthy controls, 22 Health Care Workers (HCWs) were enrolled at the Bambino Gesù Children’s Hospital.

HCWs and patients were considered eligible for the study if they were naïve to SARS-CoV-2 infection at the time of enrollment, had completed the primary schedule with the mRNA BNT162b2 vaccine, and agreed to receive the 3^rd^ dose.

In patients with CVID, the first booster (3^rd^ dose) dose was performed in September 2021, six months after completing the full primary immunization schedule. In HCWs, the first booster dose was performed nine months after the last dose of the vaccine. At the time of the booster in Italy, only the monovalent vaccine was available and approved for use.

As prescribed for patients with IEIs by the Italian Agency of Drugs (AIFA), the second booster dose (4^th^ dose) of the BNT162b2 vaccine was offered in March 2022 only to those participants who remained free from SARS-CoV-2 infection since the last vaccine administration. Patients were tested for SARS-CoV-2 infection by RT-PCR on the nasopharyngeal swab (NPS) every time they attended a hospital site, in case of positive household contacts irrespective of symptoms, and upon onset of symptoms possibly related to COVID-19. The duration of the viral shedding was evaluated by recording the dates of the first positive and first negative NPS. In patients with SARS-CoV-2 infection, we recorded COVID-19 severity [scored according to WHO stage (38)], hospitalization, vaccination status, and SARS-CoV-2 treatments).

**Figure 1:**
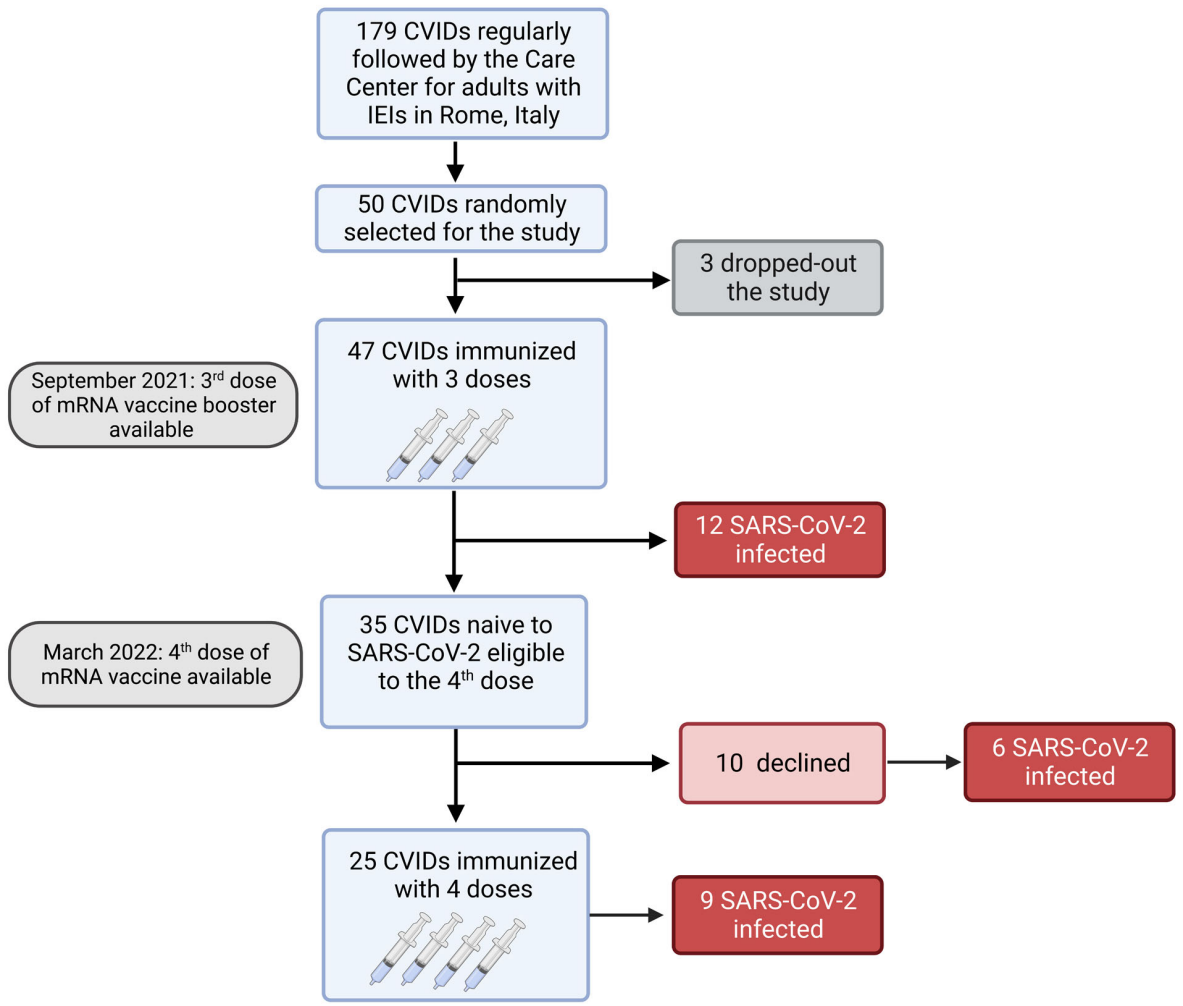
Flow chart. Observational study on 47 adults with CVIDs naïve to SARS-CoV-2 infection immunized with the booster mRNA BNT162b2 vaccine.

Blood samples were obtained 10 days after the 3^rd^ dose (post 3^rd^), six months apart on the day of the 4^th^ dose (pre 4^th^), and 10 days after the 4^th^ dose (post 4^th^). For those who were infected after the 3^rd^ dose, blood samples were collected one month after recovery and separately analyzed. For those who refused the 4^th^ dose of vaccine, blood samples were collected six months after the booster immunization and then included in the analysis as pre 4^th^ values (**Figure 1**). During the study, participants were allowed to continue their treatments, including IgRT as standard therapy for the underlying antibody deficiency. At the study time, all IgRT brands in use did not contain anti-S1 IgG antibodies (39, 40). For each participant, clinical data were collected, including age, immunoglobulin levels at IEIs diagnosis, and the number of IEI-related disorders. We defined CVIDs with immune-dysregulation phenotype as having at least one of the following: systemic autoimmunity or autoimmune cytopenia, enteropathy, lymphoproliferative disorders, and lymphoid malignancy or solid cancer. Chronic Lung Disease (CLD) was defined as having at least one of the following: Chronic Obstructive Pulmonary Disease diagnosed according to GOLD guidelines (41), Interstitial Lung Disease or Bronchiectasis (based on CT scan imaging), and Granulomatous and Lymphocytic Interstitial Lung Diseases (GLILD) (based on CT scan imaging and histologically confirmed) (42). Immunological laboratory data collected at baseline included complete blood count (CBC) and immunoglobulin serum levels.

Following the actual position of IUIS (1), eight patients displaying complex clinical phenotypes (mainly immune dysregulation) underwent molecular testing to rule out monogenic forms of humoral immunodeficiencies. A custom-targeted Next Generation Sequencing (NGS) panel of 53 genes whose variants are associated with CVIDs, and humoral immunodeficiency was performed (**Supplementary Table 1**). As targeted genetic testing did not identify known or potential molecular bases for monogenic immunodeficiency in the selected patients, all cases in the present cohort were retained.

The study was approved by the Ethical Committee of the Sapienza University of Rome (Prot. 0521/2020, July 13, 2020) and performed in accordance with the Good Clinical Practice guidelines, the International Conference on Harmonization guidelines, and the most recent version of the Declaration of Helsinki.

### 2.2 Humoral response

Humoral response to vaccination was assessed quantifying the anti-S/Receptor Binding Domain (RBD)-IgG by a commercial chemiluminescence microparticle antibody assay (ARCHITECT SARS-CoV-2 IgG II Quantitative, Abbott Laboratories, Wiesbaden, Germany) according to the manufacturer’s protocol (Architect^®^ i2000sr Abbott Diagnostics, Chicago, IL). As reported in the leaflet, anti-RBD-IgG were expressed as binding arbitrary units (BAU)/mL and values were considered positive when ≥7.1. To evaluate the neutralizing activity of vaccine induced antibodies, microneutralization assay (MNA) was performed as previously described, using both SARS-CoV-2 Wuhan (Lineage A, GISAID Accession Number: EPI_ISL_410545) and Omicron (Lineage BA.5, GISAID Accession Number: EPI_ISL_16848123) as challenging viruses (43). Briefly, heat-inactivated sera serially titrated in 8 two-fold dilutions in duplicate (starting dilution 1:10). Equal volumes of serum and medium containing 100 TCID_50_ SARS-CoV-2 Lineage A or Lineage BA.5 were mixed and incubated at 37°C for 30 min. Serum-Virus mixtures were then added to sub-confluent Vero E6 cell monolayers and incubated at 37°C and 5% CO_2_. After 72 hours, microplates were observed by light microscope for the presence of cytopathic effect (CPE). To standardize inter-assay procedures, positive control samples showing high (1:160) and low (1:40) neutralizing activity were included in each assay session. The highest serum dilution inhibiting at least 90% of the CPE was indicated as the neutralization titer and was expressed as the reciprocal of serum dilution (MNA90).

### 2.3 Cell isolation and cryopreservation

Peripheral blood mononuclear cells (PBMCs) were isolated by Ficoll Paque™ Plus 206 (Amersham PharmaciaBiotech) density-gradient centrifugation and immediately frozen and stored in liquid nitrogen until use. The freezing medium contained 90% Fetal Bovine Serum (FBS) and 10% DMSO.

### 2.4 Detection of antigen-specific B cells

For the detection of SARS-CoV-2 specific B cells, a biotinylated protein spike was individually multimerized with fluorescently labeled streptavidin as previously described (17, 25, 44). Separate aliquots of recombinant biotinylated Spike or RBD were mixed with streptavidin BUV395, streptavidin PE or streptavidin FITC (BD Bioscience) at 25:1 ratio 20:1 ratio and 2.5:1 ratio respectively. We used two different streptavidin-conjugated fluorochromes, one with a very high brightness index (PE) and the other with a moderate brightness index (BUV395), to be able to distinguish low-affinity MBCs (only visible with a very bright fluorochrome such as PE) from high-affinity MBCs (double positive for PE and BUV395). Streptavidin PE-Cy7 (BD Bioscience) was used as a decoy probe to gate out SARS-CoV-2 antigen non-specific streptavidin-binding B cells. The antigen probes individually prepared as above were then mixed in Brilliant Buffer (BD Bioscience). 4x10^6^ previously frozen PBMC samples were prepared and stained with the antigen probe cocktail containing 100 ng of spike per probe (total 200 ng), 27.5 ng of RBD, and 2 ng of streptavidin PE-Cy7. After one step of washing, surface staining with antibodies was performed in Brilliant Buffer at 4°C for 30 min.

B cell subsets were identified based on the expression of CD19 BV786, CD27 BV510, CD24 BV711, and CD38 BV421 markers by flow-cytometry purchased from BD Bioscience. Naïve B cells were identified as CD19+CD24+CD27-, transitional B cells were gated as CD19+CD24++CD38++, and atypical MBCs as CD19+CD24-CD27-CD38-. MBCs were defined as CD19+CD24+CD27+, and anti-IgM APC (Jackson ImmunoResearch) will be used to discriminate IgM+ form switched (IgM-) MBCs. Spike-specific MBCs were defined as low-affinity (positive for PE, S+) or high-affinity (double positive for PE and BUV395, S++) (**Supplementary Figure 1A**).

Among S+ and S++ MBCs, IgM and RBD expressions were evaluated (**Supplementary Figure 1B**).

Stained PBMC samples were acquired on FACs LSRFortessa (BD Bioscience). At least 2x10^6^ cells were acquired and analyzed using Flow-Jo10.8.1 (BD Bioscience). Phenotype analysis of antigen-specific B cells was only performed in subjects with at least 10 cells detected in the respective antigen-specific gate. Blank was determined in unexposed donors, before vaccination. LOB (limit of blank) was set as the mean of the blank + 1.645xSD. LOD (limit of detection) as the mean of the blank + 2xSD.

### 2.5 T-cell functional studies

PBMCs isolated from CVIDs patients were cultured in 96-well cell plates at 1x10^6^ cells/well concentration in RPMI 1640 culture medium containing 5% AB serum. PBMCs were cultured at 37°C, 5% CO_2_, with CytoStim^®^ (from the SARS-CoV-2 Prot_S PBMC Kit, Miltenyi, Biotech) for a total of six hours. After two hours of incubation, Brefeldin A (SARS-CoV-2 Prot_S PBMC Kit, Miltenyi, Biotech) was added to the cells to inhibit the transport of proteins to the cellular membrane. Cells were fixed, permeabilized, and stained with CD3 APC, CD4 Vio B515, CD8 Vio Green, CD154 (CD40L) APC Vio 770, IFNγ PE, and TNFα PE-Vio770 (SARS-CoV-2 Prot_S PBMC Kit protocol, Miltenyi Biotech) with the addition of CD45RO BUV395(BD Biosciences).

T cells were gated as CD3+ and divided into CD3+CD4+ and CD3+CD8+ T cells. Naïve T cells were gated as CD3+CD4+CD45RO- (or CD8+) and memory T cells were identified as CD3+CD4+CD45RO+ (or CD8+). The CD40L, IFNγ, and TNFα were evaluated on CD4 and CD8 memory T cells. Cells were acquired on a BD FACSymphony A5™ and data were analyzed with FlowJo ver. 10.8.1 (BD Bioscience).

### 2.6 B cells and T cells unbiased population identification

FCS files were analyzed using the FlowJo ™ v10.8.1 software (BD, Biosciences). Prior to analysis, all samples were normalized to equal cell numbers. For each of the four groups of patients, 5 randomly selected samples were concatenated in a single FCS file. Equal numbers of live CD19+ B cells or CD3+CD4+ T cells or CD3+CD8+ T cells were analyzed. Data were partitioned into clusters in high dimensional space by using the X-Shift plugin algorithm (45), and visualized after performing dimensionality reduction using the Optimized t-distributed stochastic neighbor embedding (opt-SNE). Marker expression and frequency across different clusters were then assessed using the heatmaps generated by the Cluster explorer plugin (FlowJo ™ v10.8.1 software, BD, Biosciences).

### 2.7 Genetic analysis

Sequencing experiments were performed on the Ion Torrent S5 platform (Thermo Fisher Scientific). Sequence data analysis was performed with the Ion Reporter v5.18 software (Thermo Fisher Scientific). Variant validation was performed by Sanger sequencing on the 3730 Genetic Analyzer platform (Thermo Fisher Scientific). Variants with a minor allele frequency >0.01 were filtered out. Only nonsynonymous variants and splice site variants were retained. A visual analysis of the reads including candidate variants mapped against the reference genome (version GRCh37/hg19) was performed on the Integrative Genome Viewer software (IGV, https://software.broadinstitute.org/software/igv/). Variants were classified according to the American College Of Medical Genetics And Genomics (ACMG) guidelines (46). Data are available at http://www.ncbi.nlm.nih.gov/bioproject/967329.

### 2.8 Statistical analysis

Data were analyzed by GraphPad Prism 9 version 9.3.1 and Statistical Package for Social Sciences version 15 (SPSS Inc). The data were first tested for normality and homoscedasticity using Shapiro-Wilk and Levene’s tests and since the assumptions were violated, non-parametric tests were used for the analysis.

Demographics were summarized with descriptive statistics (median and IQR for continuous values). Immunological and clinical variables were compared between the different study times. A univariate analysis assessed the impact of variables of interest. The tests of normalcy variables did not follow normal distribution according to the results of the Kolmogorov–Smirnov and Shapiro–Wilks tests. As a result, a nonparametric approach was used to analyse the data, the Wilcoxon matched pair signed-rank test or the two-tailed Mann–Whitney U-test were used. Categorical variables were compared by Chi-square test. Differences were deemed significant when *P* < 0.05).

## 3 Results

### 3.1 Patients

Among 179 patients with CVIDs, 50 were randomly selected and agreed to participate to the study. Three patients dropped-out and 47 CVIDs patients [median age 53.5 years (IQR 45.7-66), females 28 (58.3%)], immunized with the 3^rd^ dose of the mRNA BNT162b2 vaccine, were included in the analysis. Six months apart, the 4^th^ dose was administered to 25/47 patients, because 12 participants were infected by SARS-CoV-2 and 10 patients refused the additional booster doses (**Figure 1**).

### 3.2 Serum anti-S1 IgG after the first booster vaccine dose

**Figure 2:**
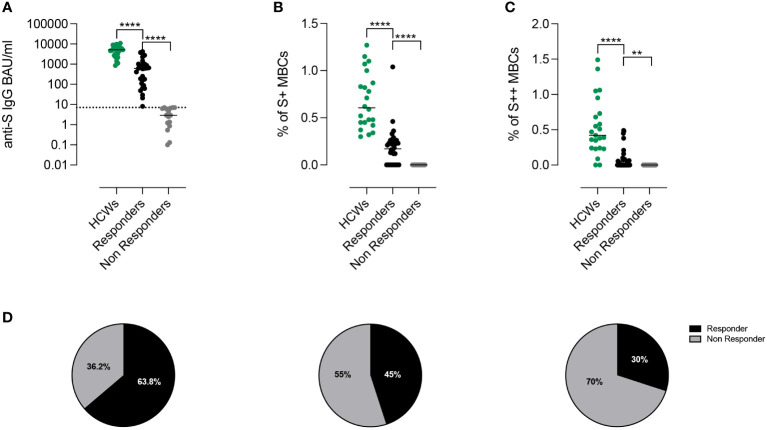
Proportion of responders after third dose of BNT162b2 accordingly to Anti-S1 IgG antibodies and spike-specific memory B cells. **(A)** Individual anti-S1 IgG values in HCWs (green) and in patients with CVIDs. The values measured in the patients with anti-S1 IgG above the cut-off value (dotted line) are shown in black and those below in gray. Bars indicate median values. **(B, C)** Frequency of low (S+) and high-affinity (S++) MBCs in HCWs (green) and in *Responders* and *Non-Responders* CVIDs patients. **(D)** The pie charts depict the percentage of *Responders* or *Non-Responders* by considering only the presence of antibodies above the cut-off (left), by combining the presence of antibodies and S+ MBCs (centre), or by compounding antibodies S+ and S++ MBCs (right). Non-parametric Mann–Whitney t-test was used to evaluate statistical significance. Two-tailed P value significances are shown as **p< 0.01, ****p< 0.0001.

After the 3^rd^ dose, all HCW controls (median age 54.8 years, IQR 43.8-61.9) had measurable levels of serum anti-S1 IgG Abs. In contrast, specific IgG were above the cut-off value in 30/47 (63.8%) CVIDs patients (median 617.6 BAU/ml, IQR 99.44-967.8) (**Figure 2A**). We separately show the antibody levels in CVIDs patients that exceeded (*Responders, R*) or not (*Non-Responders, NR*) the cut-off value (7.1 BAU/ml). CVIDs patients able to seroconvert had a highly variable response with a significantly lower antibody concentration than control HCWs (**Figure 2A**). The data suggest that, even when we exclude *Non-Responders* CVIDs patients from the analysis, our cohort includes individuals with different capacities to mount an antibody response.

### 3.3 Peripheral blood Spike-specific memory B cells after the first booster vaccine dose

High specificity and affinity are the most important characteristics of MBCs, generated by the adaptive immune system in response to infection or vaccination (25, 44). In control HCWs, low-affinity (S+) MBCs can be detected before vaccination, whereas high-affinity (S++) MBCs are produced only after the 2^nd^ vaccine dose (44). S+ MBCs are mostly of IgM isotype and S++ are switched MBCs (**Supplementary Figure 1B**). The RBD specificity is absent among S+ MBCs. In contrast, 20-40% of the S++ MBCs bind RBD and are of switched isotype (**Supplementary Figure 1B**).

We measured S+ (**Figure 2B**) and S++ MBCs (**Figure 2C**) ten days after the 3^rd^ dose in patients who were defined as *Responders* or *Non-Responders* based on the level of specific Abs. Overall, as compared to control HCWs, CVID patients had significantly lower frequencies of S+ and S++ MBCs. S+ MBCs were detectable in 45% (21/47) of the CVIDs patients (**Figure 2B**) and S++ MBCs in 30% (14/47) of the cases (**Figure 2C**).

Thus, depending on the biological read-out of efficacy used, a different percentage of patients can be classified as *Responders* or *Non-Responders* (63.8%, based on antibody levels, vs 45%, based on S+ MBCs vs 30%, based on S++ MBCs) (**Figure 2D**).

**Figure 3:**
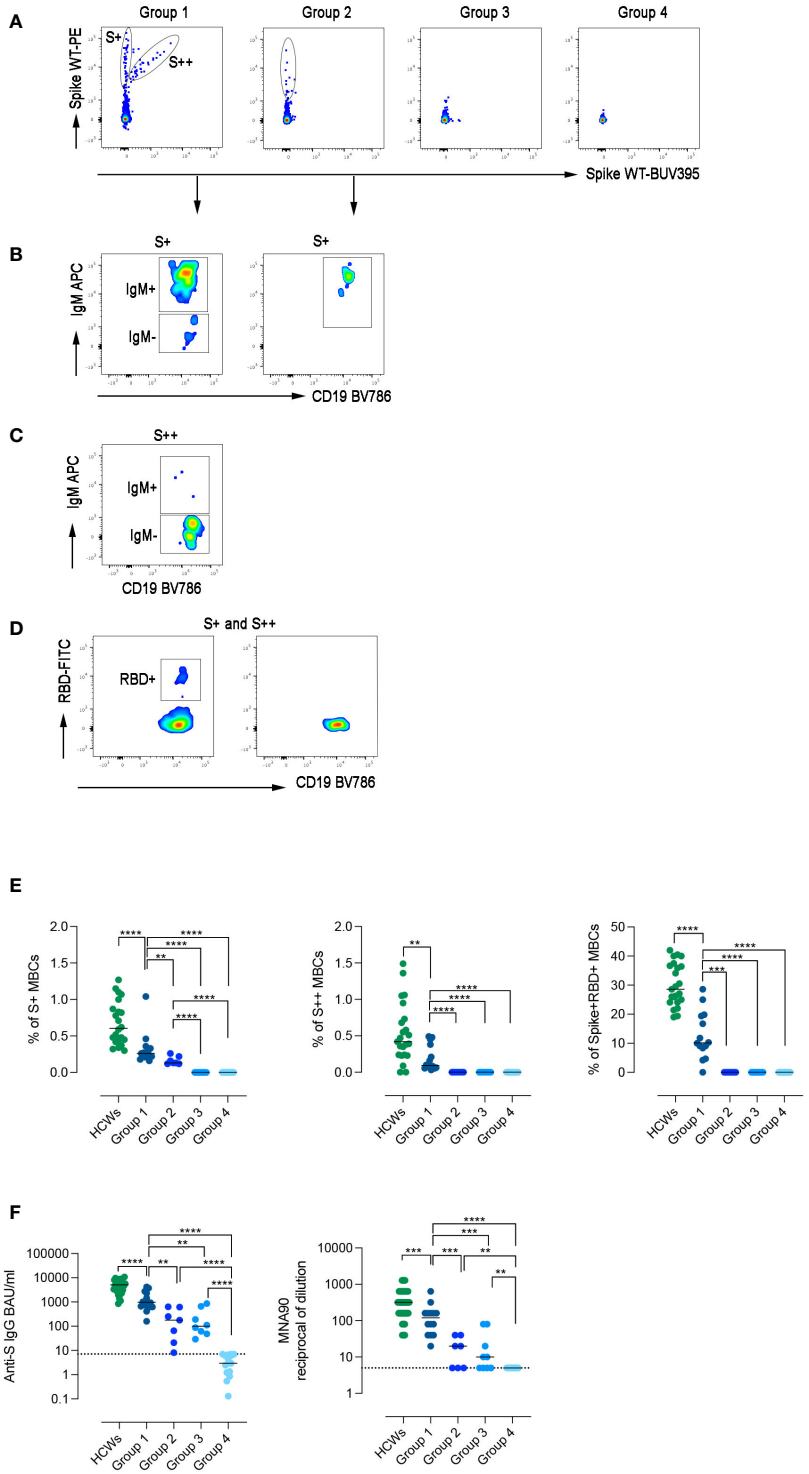
Specific immune response to SARS-CoV-2. **(A)** Spike-specific MBCs with low (S+) and high (S++) binding capacity are shown in patients representative of the four groups. **(B)** S+ MBCs, detectable only in patients of groups 1 and 2, are mostly of IgM+ isotype. **(C)** S++MBCs, found only in group 1, are IgM- MBCs. **(D)** RBD-specific MBCs were only detectable in group 1. **(E)** Dot plots represent the frequency of S+, S++, and RBD+ MBCs in the four groups of CVIDs patients and HCWs. **(F)** The plots show S1-specific IgG and neutralizing antibody titers in serum samples of HCWs (in green), and CVIDs patients. Dot line indicates the cut-off value. Bars indicate the median. Non-parametric Mann–Whitney t-test was used to evaluate statistical significance. Two-tailed P value significances are shown as **p< 0.01, ***p< 0.001, ****p< 0.0001.

Among CVIDs patients who could be considered *Responders* based on antibody levels, we identified three different types of immune responses (**Figure 3A**; **Table E2**), which were used to define functional groups of patients. Fourteen patients (group 1) generated both S+ and S++ MBCs, 7 patients (group 2) generated only S+ MBCs and 9 patients (group 3) had no spike-specific MBCs. The 17 *Non-Responder* patients, lacking both Abs and spike-specific MBCs, were assigned to group 4 (**Figure 3A**).

High-affinity MBCs are generated in the germinal centers, with the help of T cells, thanks to the combined mechanisms of somatic hypermutation and antigen selection (47). Only a minority of CVIDs patients have high-affinity S++ MBCs suggesting that only patients of this group can perform all the functions required to complete the adaptive immune response.

Although group 1 patients generated an immune response composed of S+, S++, and RBD+ MBCs (**Figures 3B–D**), all frequencies were significantly lower compared to HCWs (**Figure 3E**).

In group 2, only S+ IgM MBCs were detectable (**Figure 3B**), whereas patients of groups 3 and 4 failed to generate any spike-specific MBCs.

Serum anti-S1 IgG levels differed in the four groups, being significantly higher in group 1 than in groups 2 and 3 (p=0.0016 and p=0.0012, respectively) (**Figure 3F**). In group 1, anti-S levels were significantly lower than in HCWs and had a reduced neutralizing activity against wild-type SARS-CoV-2. Neutralizing Abs titers were significantly lower in group 2 and 3 and absent in group 4 (**Figure 3F**).

### 3.4 The immune response after the 4^th^ vaccine dose

Six months after the 3^rd^ dose, the 4^th^ dose was administered to 25 patients. Twelve patients did not receive the second booster because they were infected by SARS-CoV-2 after the 3^rd^ dose (**Figure 1**) and were not included in the longitudinal study.

**Figure 4:**
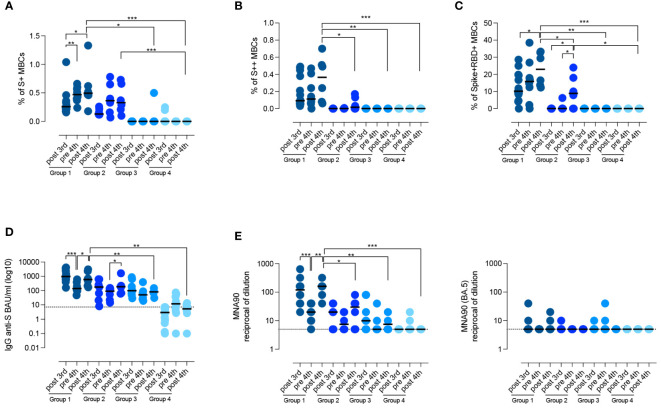
Persistence of the specific response against SARS-CoV-2. **(A–D)** Graphs show the frequency of spike-specific S+ MBCs **(A)** S++ MBCs **(B)**, RBD+ MBCs **(C)**, the concentration of anti-S1 IgG **(D)** and the neutralizing antibody titers for the wild type and the BA.5 variant **(E)**, after the 3^rd^ dose, before and 10 days after the 4^th^ dose in the four groups of CVIDs patients. Medians are shown as bars and the dotted line indicates the cut-off. Non-parametric Wilcoxon matched pair signed-rank test and Mann–Whitney t-test were used to evaluate statistical significance. Two-tailed P value significances are shown as *p<0.05, **p< 0.01, ***p< 0.001.

After the 4^th^ vaccine dose, in group 1, the frequency of S+, S++ RBD-specific MBCs significantly increased (p=0.0386, p=0.0122, p=0.0245, respectively) (**Figures 4A–C**). In group 2, the frequency of S+ MBCs did not change, but few S++ and RBD-specific MBCs were generated in four patients (**Figures 4B–D**). In one patient of group 3, S+ MBCs became detectable after the 4^th^ dose (**Figure 4A**).

In the pre-4^th^ dose serum sample, anti-S1 IgG levels were still detectable in all patients who had produced Abs before (groups 1, 2, and 3) (**Figure 4D**). In response to the 4^th^ dose, anti-S1 IgG increased in groups 1 and 2 (p=0.0500), but not in groups 3 and 4 (median 5.35 BAU/ml, IQR 2.67-10.7) (**Figure 4D**). Thus, only patients who generated spike-specific MBCs were able to secrete Abs in response to the booster.

Virus neutralization after the 4^th^ dose confirmed that patients of group 1 had a more effective response than patients of groups 2, 3, and 4, resulting in the generation of Abs able to prevent viral infection *in vitro* (p=0.0152, p=0.0095, and p=0.0006 respectively) (**Figure 4E**). In immunocompetent subjects, administration of the 3^rd^ and 4^th^ dose induces the production of Abs able to also neutralize the omicron variants, independently of previous exposure to the novel strains (48). Broadening of the immune response did not occur in CVIDs patients as they were all unable to produce Abs with neutralizing activity against the omicron variant BA.5 (**Figure 4E**).

### 3.5 Isotype of Spike-specific MBCs

**Figure 5:**
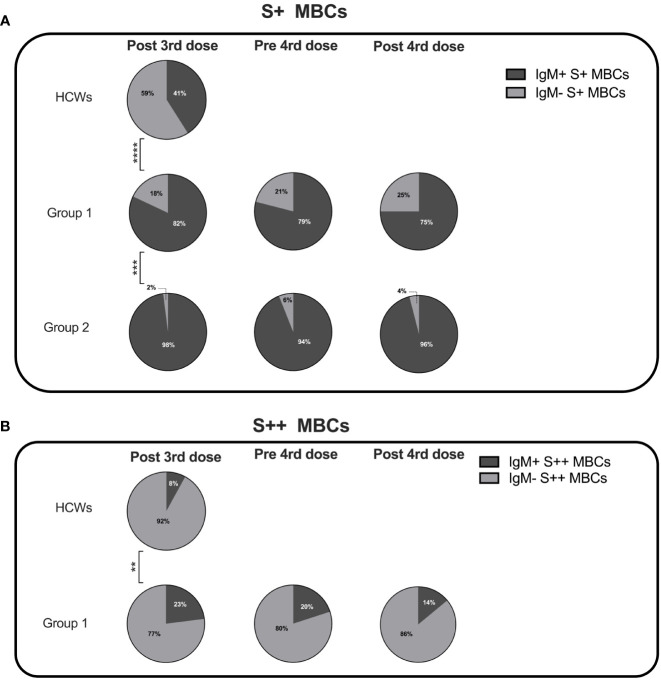
Percentage of IgM+ and IgM- specific MBCs. Pie charts depict the percentage of IgM+ and IgM- S+ **(A)** and S++ MBCs **(B)** in HCWs, and groups 1 and 2 CVIDs patients at the time points of analysis. MBCs were always undetectable in group 3 and group 4. Categorical variables were compared by Fisher exact test. Level of significance: **p< 0.01, ***p<0.001, ****p< 0.0001.

We compared the isotype of MBCs generated in response to vaccination in HCWs and CVIDs patients of groups 1 and 2. In HCWs, S+ MBCs were half of IgM and half of switched isotypes, whereas S++ MBCs were almost all switched (**Figures 5A, B**).

Class switching appeared to be less effective in CVIDs patients since both in groups 1 and 2 S+ MBCs were mostly of IgM isotype (**Figure 5A**). S++ MBCs, detectable only in patients of group 1 at all-time points, were in the majority switched MBCs but remained significantly lower than in the controls (**Figure 5B**).

### 3.6 B-cell phenotype analysis

**Figure 6:**
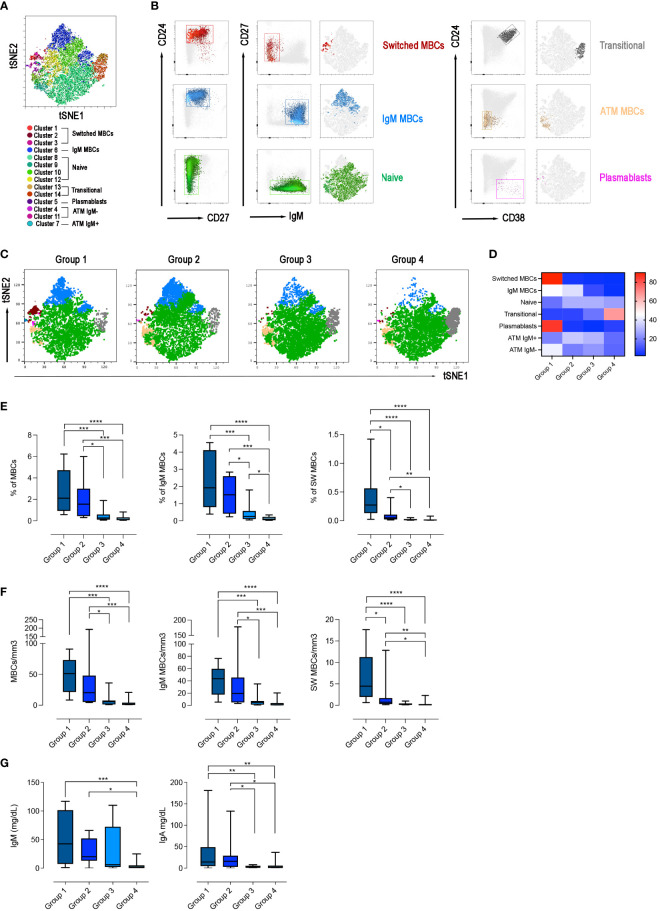
B-cell population in the 4 groups. **(A)** X-Shift B-cell cluster sets originated from the four concatenated groups and overlaid onto the Opt-SNE map, each cluster is indicated by a color. Based on the relative expression level of surface markers calculated by X-shift, we identified naïve B cells (clusters 11, 12, 13 and 15), switched MBCs (clusters 1, 2 and 3), IgM MBCs (cluster 6), transitional B cells (clusters 13 and 14), plasmablasts (cluster 5), and atypical MBCs IgM- (clusters 4 and 11) and IgM+ (cluster 7). **(B)** Expression of CD24, CD27, CD38 and IgM for population characterization and identification. For sake of simplicity, we represent the same population as one cluster with one color. **(C)** Merged Opt-SNE plots for each group with relative X-Shift cluster sets overlaid onto the Opt-SNE map. **(D)** Heat map depicts the cluster sets abundancy (%) in the four groups. Bar plots depict the frequency **(E)** and the absolute numbers **(F)** of MBCs (CD19+CD24+CD27+), IgM (IgM+), switched (IgM-) MBCs, in the four groups of CVIDs patients. The frequency of the B cell populations was evaluated in the lymphocyte gate. **(G)** Serum levels of IgM and IgA in the four groups. Non-parametric Mann–Whitney t-test was used to evaluate statistical significance. Two-tailed P value significances are shown as *p<0.05, **p< 0.01, ***p< 0.001, ****p< 0.0001.

We asked the question of whether the four groups of CVIDs patients, defined by the response to SARS-CoV-2 vaccination, correspond to distinct B-cell phenotypes. On samples obtained after the 3^rd^ dose, we performed an unbiased analysis of CD19+ B cells using optimized t-stochastic neighbor embedding (Opt-SNE) to reduce dimensionality, followed by graph-based clustering analysis (X-shift). We identified 14 B-cell clusters in the four concatenated groups (**Figure 6A**). The B cell clusters were analyzed by marker expression to identify the different B cell populations resulting in the definition of seven major clusters (**Figure 6B**). The relative X-Shift cluster sets were calculated for each of the four groups (**Figure 6C**). The main differences among the groups were caused by the distribution of MBCs.

We found that switched MBCs were present only in group 1, whereas IgM MBCs were identified in groups 1 and 2 (**Figures 6C, D**). Atypical MBCs which have been shown to be increased in autoimmune disease, chronic viral infection, and in a group of CVIDs patients (49, 50), were detected at low frequencies in all groups. Plasmablasts were found only in group 1 and transitional B cells were increased in group 4 (**Figures 6C, D**).

Based on the populations identified by cluster analysis, we calculated the frequency and absolute numbers of the identified populations in all patients of the four groups. Frequency and absolute numbers of MBCs and IgM MBCs were within the normal range in groups 1 and 2 but switched MBCs were significantly reduced in group 2 compared to group 1 (frequency: p=0.0159; absolute numbers: p=0.0297, **Figures 6E, F**). Patients of group 3 had significantly fewer MBCs, both of IgM and switched isotypes, compared to groups 1 and 2 (**Figures 6E, F**). Patients of group 4 were lymphopenic and CD19+ B cells were significantly reduced (**Supplementary Figures 2A, B**). Despite the reduction of total B cells, transitional B cells, the most immature cell type found in the peripheral blood, were present at normal frequency and numbers indicating a normal output from the bone marrow. In contrast, mature-naive B cells and MBCs were strongly reduced, both in frequency and absolute numbers (**Supplementary Figures 2A, B**, **6E, F**). In groups 1, 2 and 3 transitional and mature-naive B cells were within the normal values (**Supplementary Figures 2A, B**). The frequency and the absolute number of atypical MBCs were comparable in the four groups of patients (**Supplementary Figures 2A, B**).

In summary, group 1 patients had a B cell phenotype similar to that of control HCWs, and all B-cell populations were detected at normal frequencies. Patients of group 2 had normal numbers of IgM MBCs but were characterized by a strong reduction of switched MBCs. Patients in group 3 had no MBCs. Patients of group 4 had low total B-cell numbers with a significant reduction of mature-naive B cells and the absence of MBCs.

In line with the description of the B-cell phenotype, IgM serum levels measured at the study time were significantly reduced in group 4 compared with groups 1 and 2 (p=0.0001 and p=0.0238, respectively) and IgA levels were significantly reduced in groups 3 and 4 compared to group 1 (p=0.0052, 1 *vs* 3; p=0.0018, 1 *vs* 4) and group 2 (p=0.0428, 2 *vs* 3; p=0.0500, 2 *vs* 4) (**Figure 6G**).

### 3.7 T cell phenotype and function in the four groups of CVIDs patients

T cells in groups 1 and 2 were present in normal frequency and absolute numbers, while total CD4+, CD4+ memory (CD45RO+) and naïve CD4+ (CD45RO-) were low in group 4 (**Supplementary Figure 3**). One patient of group 3 (11%) and 6 of group 4 (35%) had a severe reduction in CD4+ (<200 cells/mm3) and/or naïve CD4+ naive (20 cells/mm3), thus fulfilling the criteria for the diagnosis of Late-Onset Combined Immunodeficiency (LOCID) (51). The frequency and the absolute number of total CD8+ and both memory and naïve CD8+ were comparable in all groups (**Supplementary Figure 3**).

**Figure 7:**
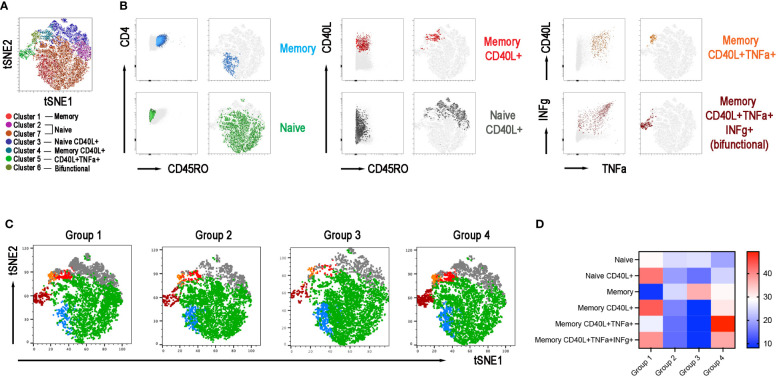
CD4+ T-cell population in the 4 groups. **(A)** X-Shift B-cell cluster sets originated from the four concatenated groups and overlaid onto the Opt-SNE map, each cluster is indicated by a color. Naïve T cells were identified in two clusters 2 and 7 and were merged together. **(B)** Expression of CD45RO, CD40L, IFNγ, and TNFα for population characterization and identification. **(C)** Merged Opt-SNE plots for each group with relative X-Shift cluster sets overlaid onto the Opt-SNE map. **(D)** Heat map depicts the cluster sets abundancy (%) in the four groups.

The adaptive response to vaccination requires the collaboration of B cells with CD4+ T cells in the GC. We have demonstrated before that switched MBCs can be only generated in individuals with functional GCs (33). IgM MBCs, instead, have been also detected in individuals with severe immunodeficiencies, lacking T cells or the costimulatory molecule CD40 Ligand (CD40L) (33). Because of their importance in the GC reaction and generation of immune memory, we analyzed the function of CD4+ T cells activated by polyclonal stimulation in samples obtained after the 3^rd^ dose. We evaluated their phenotype and ability to respond with upregulation of the CD40L and secretion of IFNγ and TNFα. The Opt-SNE map identified 7 clusters corresponding to naïve and memory CD4+ T cells, that expressed or not the CD40L and secreted TNFα and/or IFNγ (**Figures 7A, B**). The relative X-Shift cluster sets were calculated for each of the four groups of CVID patients (**Figure 7C**). As shown by the heatmap, the function of naïve and memory T cells was maintained in group 1, slightly altered in group 4, but severely impaired in groups 2 and 3 (**Figure 7D**). All the data obtained with unbiased analysis were confirmed by a bias analysis (see **Supplementary Figure 4**). CD40L was significantly diminished in all groups compared to group 1. In groups 2 and 3, also the mean fluorescence intensity (MFI) for the CD40L, which is proportional to the number of expressed molecules, was lower than in group 1. Among CD4+ T cells, those that express the CD40L after stimulation also secrete IFNγ and/or TNFα and were significantly reduced in patients of groups 2 and 3 (**Supplementary Figure 4**).

We also performed an unsupervised analysis of CD8+ T cells phenotypes following polyclonal stimulation. The Opt-SNE map identified 7 clusters corresponding to naïve and memory CD8+ T cells, that secreted or not TNFα and/or IFNγ and expressed or not the CD40L (**Supplementary Figure 5A**). The relative set of X-Shift clusters for each group were calculated (**Supplementary Figure 5B**).

The heatmap shows that also on CD8+ cells the expression of the CD40L was severely impaired in groups 2 and 3, thus confirming the alteration of this pathway (**Supplementary Figure 5C**). Analyzing the data with a classical gating strategy we confirmed that CD8+ cells expressing CD40L were significantly diminished in groups 2, 3, and 4 compared to group 1. In groups 2 and 3, also the MFI was lower than in group 1 (**Supplementary Figure 5D**).

IFNγ and TNFα are not co-expressed with CD40L in CD8+ cells. The frequency of memory CD8+ cells able to secrete IFNγ was significantly upregulated in patients of groups 2, 3, and 4 compared to those of group 1 and the frequency of CD8+TNFα+ cells were significantly increased in patients of groups 2 and 4 compared to group 1 (**Supplementary Figure 5E**).

### 3.8 CVIDs clinical phenotypes

**Figure 8:**
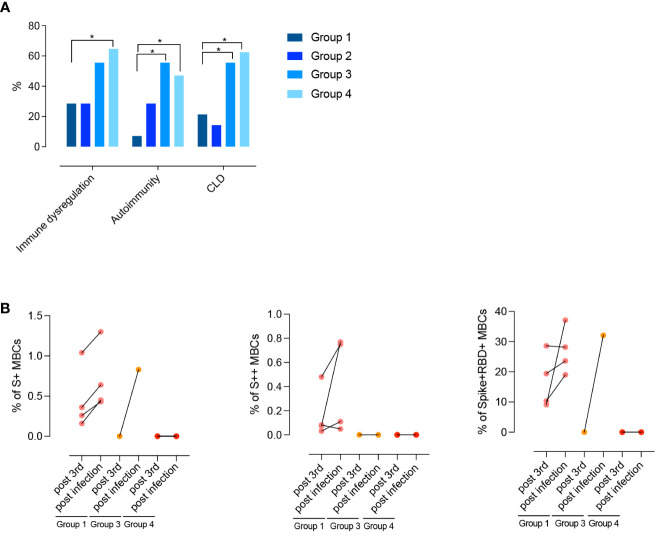
**(A)** Clinical phenotypes (immune dysregulation, autoimmunity, and CLD) of CVIDs patients grouped according to post-immunization S+ and S++ MBCs response and anti-S1 IgG. **(B)** Paired dot plots depict the S+, S++ and RBD+ MBCs measured before and after an infection in CVID patients who had COVID-19 after 3^rd^ dose. Categorical variables were compared by Fisher exact test. Level of significance: *p<0.05.

Differences between groups are mirrored by the clinical and immunological characteristics of participants (**Table 1**; **Figure 8A**). Groups 1 and 2 had a less severe clinical phenotype than groups 3 and 4, with a significantly lower frequency of patients with signs of immune dysregulation, lower prevalence of chronic lung disease (**Figure 8A**), lower cumulative number of IEI-related disorders, and absence of lymphopenia (<1000 cells/mm3) (**Table 1**). These observations confirm that the presence of MBCs is associated with a less severe course of clinical disease (52). The inability of patients of group 2 to generate high-affinity MBCs, supports the notion that IgM MBCs have the function of systemic and mucosal first-line protection (34, 53), but do not represent the selected product of adaptive immunity.

**Table 1:** Clinical characteristics and immunological data of 47 CVIDs patients.

	All CVIDs n=47	Group 1 (S1 IgG+/S+ and S++ MBCs+) n=14 (30%)	Group 2 (S1 IgG+/S+ MBCs+) n=7 (15%)	Group 3 (S1 IgG+/S+ MBCs-) n=9 (19%)	Group 4 (S1 IgG-/S+ MBC-) n= 17 (36%)	p values <0.05
Sex. Male n (%)	28 (59.6)	8 (57)	4 (57)	6 (66.7)	10 (58.8)	–
Age. median (IQR)	53 (45-66)	54 (46-71)	53 (46-59)	45 (38-60)	56 (50-65)	–
Age at IEI diagnosis median (IQR)	38 (32-51)	40 (35-57)	37 (29.5-49)	39 (30-44)	38 (33-51)	–
Serum IgG at IEI diagnosis median (IQR)	307 (166-433)	440 (456-583)	144 (28-259)	320 (120-396)	240 (166-375)	gr 1 vs 4 p<0.0001 gr 1 vs 3 p=0.002 gr 1 vs 2 p=0.001
Serum IgA at IEI diagnosis median (IQR)	20 (6-28)	47 (6-103)	13 (8-16)	25 (16-26)	11 (1-27)	gr 1 vs 4 p=0.0018 gr 1 vs 3 p=0.0052 gr 2 vs 4 p=0.0489 gr 2 vs 3 p=0.0428
Serum IgM at IEI diagnosis median (IQR)	17 (3-42)	50 (25-59)	18 (3-52)	16 (3-36)	5 (0-17)	gr 1 vs 4 p<0.0001 gr 2 vs 4 p=0.0238
Persistent lymphopenia (<1000 cell/mm3), n (%)	10 (21.3)	0	0	4 (44)	6 (35.3)	gr 1 vs 4 p=0.013 gr 1 vs 3 p=0.006 gr 2 vs 3 p=0.042
Dysregulatory- phenotype, n (%)	22 (46.8)	4 (28.6)	2 (27.6)	5 (55.6)	11 (64.7)	gr 1 vs 4 p=0.049
Autoimmunity, n (%)	16 (34.0)	1 (7)	2 (29)	5 (55.5)	8 (47)	gr 1 vs 4 p=0.015 gr 1 vs 3 p=0.010
Autoimmune cytopenias, n (%)	9 (19.1)	0 (0)	1 (14)	2 (22)	6 (35)	gr 1 vs 4 p=0.013
Lymphoma, n (%)	4 (8.5)	2 (14)	0	0	2 (12)	–
Chronic Lung Disease, n (%)	19 (41.3)	3 (21)	1 (14)	5 (55)	10 (62)	gr 1 vs 4 p=0.024 gr 2 vs 4 p=0.033
Immunosuppressive treatment, n (%)	6 (12.8)	0	1 (14)	0	5 (29.4)	gr 1 vs 4 p=0.027
CVIDs - related manifestations, median (IQR)	1 (0-2)	0 (0-1)	1 (0-1)	2 (1-3)	2 (1-3)	gr 1 vs 4 p<0.0001 gr 1 vs 3 p<0.0001 gr 2 vs 3 p=0.014 gr 2 vs 4 p=0.005

*CVIDs, Common Variable Immune Deficiencies; IEIs, Inborn Errors of Immunit; IQR, interquartile range; MBC, Memory B Cells; n, number.*

For 10/47 patients who presented with complex phenotype, monogenic forms of CVIDs were investigated by targeted gene sequence analysis (**Table E1**). No Pathogenic or Likely Pathogenic Variants were identified, and no molecular diagnosis was achieved. The NGS analysis identified one or more constitutional heterozygous variants classified as Variants of Uncertain Significance (VUS) in four patients (**Table E3**).

### 3.9 COVID-19 infection post-immunization

After receiving the 3^rd^ dose of the vaccine, 12/47 (25%) patients were infected by SARS-CoV-2. Infected patients were previously classified as being part of groups 1, 3, or 4. One month after recovery, the frequency of S+, S++, and RBD+ MBCs increased in group 1. One patient of group 3 generated S+ MBCs of IgM isotype that were able to bind RBD (**Figure 8B**). No response was detectable in patients of group 4.

Nine out of 25 (36%) of CVIDs patients were infected by SARS-CoV-2 after receiving the 4^th^ dose of vaccine, and 6/10 (60%) were infected after refusing the 4^th^ dose (p=0.436).

The vast majority (24/27) of infected patients received a SARS-CoV-2 specific treatment (monoclonal Abs or antivirals). Twenty-six patients (96%) had mild COVID-19, whereas one patient from group 4 who refused to receive the 4^th^ dose died due to the progression of lymphoma (**Table 2**).

**Table 2:** COVID-19 infection courses in enrolled patients.

Group	Sex/age	Days of qRT PCR positivity	SARS-CoV-2 treatment	Vaccine dose	Infection course
group 3	F/82	26	MoAbs	3D	mild
group 3	M/41	8	No	3D	asymptomatic
group 4	F/66	22	MoAbs	3D	mild
group 4	F/65	41	MoAbs	3D	mild
group 1	M/52	14	MoAbs	3D	mild
group 4	F/79	25	MoAbs	3D	mild
group 4	F/72	16	MoAbs	3D	mild
group 1	F/47	37	MoAbs	3D	asymptomatic
group 1	M/56	26	MoAbs	3D	mild
group 1	M/33	11	MoAbs	3D	mild
group 4	M/82	7	Antiviral	3D	mild
group 4	M/60	8	MoAbs	3D	mild
group 4	M/50	75	MoAbs	3D	mild
group 4	M/55	14	Antiviral	3D	asymptomatic
group 4	F/66	21	MoAbs	3D	asymptomatic
group 1	F/45	26	Antiviral	3D	mild
group 3	F/56	10	No	3D	mild
group 3	F/47	30	MoAbs	3D	mild
group 3	F/69	10	MoAbs	4D	mild
group 2	M/60	17	MoAbs	4D	mild
group 1	F/39	10	No	4D	mild
group 1	F/66	10	Antiviral	4D	mild
group 4	M/56	10	Antiviral	4D	mild
group 1	F/74	9	Antiviral	4D	asymptomatic
group 1	M/57	7	Antiviral	4D	mild
group 4	M/50	10	Antiviral	4D	mild
group 3	F/45	10	Antiviral	4D	mild

## 4 Discussion

We exploited the unique opportunity offered by the vaccination with a novel antigen never encountered by humans before, in a period when immunoglobulins, administered as substitution therapy, did not contain anti-SARS-CoV-2 IgG yet, to identify functional groups among CVIDs patients. Most of the published studies evaluate the vaccine response by measuring SARS-CoV- 2 spike protein-specific antibody response, however, the durability of antibody response is known to decline over months and could be severely impaired in patients with antibody deficiency. In a recent paper, authors highlight the importance of evaluating also the cellular immune response in individuals with immunodeficiency (54).

In this longitudinal study on adult CVIDs patients immunized with one or two booster doses of the Pfizer-BioNTech (BNT162b2) vaccine, we combined the analysis of S1-specific IgG and spike-specific MBCs.

We demonstrate that the measurement of specific Abs is not sufficient to consider a patient *responder* to the vaccine, because 63.8% of the patients produce S1-specific IgG, but only 30% generate high-affinity MBCs upon booster vaccination (3^rd^ dose).

Moreover, the integration of the data on S1-specific IgG level and spike-specific MBCs frequency identifies four different groups of CVIDs patients with immune impairments of increasing severity.

CVIDs patients of group 1 generated spike-specific Abs and high-affinity switched MBCs. Group 2 and group 3 patients had lower levels of specific IgG. Whereas group 2 only generated low-affinity IgM MBCs, no MBCs were detected in group 3 patients. Finally, patients of group 4 lacked both Abs and MBCs.

In normal individuals, the 3^rd^ and 4^th^ doses with a monovalent vaccine induce the production of Abs that neutralize not only the wild-type but also the BA.5 variant (55). In the CVID patients, the neutralizing activity against wild-type SARS-CoV-2 was measurable in group 1 and, to a lower extent, in group 2, but none of the patients had Abs able to neutralize BA.5. In normal individuals vaccinated against SARS-CoV-2, the antibody and MBCs repertoire broadens in time probably because of the maintained GC function (55) and the selective pressure of pre-existing SARS-CoV-2 Abs (48). This mechanism is impaired in CVIDs patients, including those of group 1.

The four functional groups of patients have distinct B-cell phenotypes. Group 1 patients have both IgM and switched MBCs, in group 2 IgM MBCs are present in normal numbers but switched MBCs are severely reduced. All MBCs are missing in group 3, and group 4 patients have low numbers of total B cells, with a significant reduction of mature-naive B cells and the absence of MBCs.

CVID clinical phenotypes are usually identified using the EUROCLASS definition which is based on the total B cell number and distribution and size of the transitional and switched MBCs populations (49).

Our work does not have the aim of developing a new classification for CVID. We defined the four groups based on their ability to generate MBCs and Abs in response to the vaccination. Our groups with different B-cell phenotypes and patterns of response to immunization would not be identified by the EUROCLASS, mainly because of the exclusion from this classification of IgM MBCs as a parameter.

CVIDs patients with normal numbers of IgM and switched MBCs have less severe disease (52). The frequency and function of peripheral IgM MBCs are predictive of the T-independent response to polysaccharide vaccines and clinical outcomes (6, 52) and, as confirmed here, in the absence of IgM MBCs, patients with CVIDs have a more severe disease with a significantly higher frequency of chronic lung disease and immune dysregulation (52). Moreover, IgM MBCs may play a crucial role in COVID-19, as monoclonal Abs derived from IgM MBCs from patient’s convalescent from the infection with the Wuhan virus are potent neutralizers not only for the wild-type strain but also for its emerging viral variants (56). Similarly, Abs derived from IgM MBCs were able to neutralize the H1N1 influenza virus to which the donor had never been exposed (57). Here we confirm that specific MBCs, able of recall responses after a renewed encounter with the antigen, are established only if switched MBCs can be generated. Thus, the first-line protection ensured by IgM MBCs is sufficient to protect patients from severe CVIDs disease in the absence of highly specific B–cell immunity.

Because of their importance in the GC reaction and generation of MBCs, we analyzed the function of CD4+ T cells activated by polyclonal stimulation. Although T cell numbers were preserved in all patients, the function of CD4+ T cells (CD40L expression and cytokines secretion) was severely impaired in patients of groups 2 and 3.

**Figure 9:**
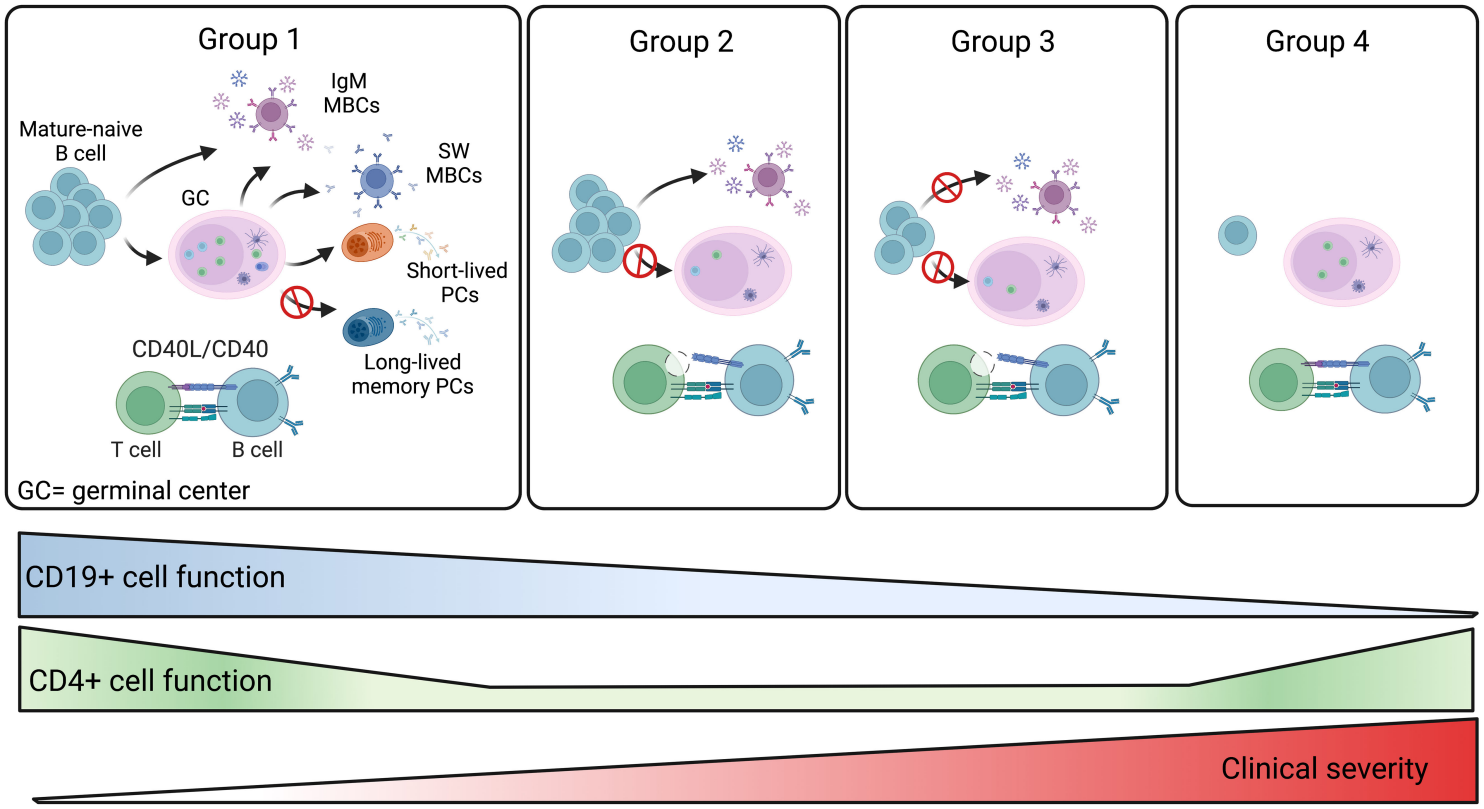
Summary of the four CVIDs groups. The memory B-cell compartment includes GC- and T-independent IgM MBCs, as well as post-GC and T-dependent MBCs of both IgM and switched isotypes. Short-lived plasmablasts (PB) are generated by recent immune responses. Long-lived plasma cells (LLPCs) maintain serum antibody levels in time. Group 1 patients are able to produce all MBCs types. Hypogammaglobulinemia may be explained by the inability to produce LLPCs in this group of CVIDs patients. For patients of groups 2 and 3, the defective CD4+ T-cell function may impair GC development. Patients of group 2 retain the ability to generate T-independent IgM MBCs, whereas all MBCs are missing in group 3. In patients of group 4, the significant reduction of all B-cell populations past the transitional B-cell stage suggests a severe defect in all B-cell functions. The severity of the clinical disease increases in groups 3 and 4.

Based on the combination of all our data we propose a model that explains the defective immune response of the four groups of patients (**Figure 9**). Group 1 patients have preserved B and T cell function and respond to vaccination. Further studies are necessary to establish whether the antibody deficiency in these patients may be explained by a defect in the generation or persistence of long-lived memory plasma cells, the population responsible for the maintenance of high-affinity and durable antibody responses (**Figure 9**). Group 2 patients produce low levels of specific IgG and low-affinity MBCs of IgM isotype that, as shown before, are generated by a T- and GC-independent mechanism (32, 33). In group 2, the impaired capacity of T cells to express the CD40L suggests that the GC reaction may be defective, thus explaining the absence of high-affinity MBCs and the impairment of class-switching (**Figure 9**). Group 3 patients may have a combined defect, with B cells unable to undergo T-independent development into IgM MBCs and T cells with insufficient expression of CD40L and IFNγ and TNFα cytokines (**Figure 9**). Finally, in group 4, a B cell-intrinsic defect may alter B cell survival and abolish their differentiation, whereas T cell function is preserved (**Figure 9**).

CVIDs is considered a “blurry” disorder (58) including patients with different, yet unidentified genetic defects. To avoid bias of inclusion, in patients with complex/atypical phenotypes, we excluded monogenic forms of CVIDs and phenocopies by sequencing a panel of genes involved in monogenic disease with defects in humoral immunity.

NGS approaches will give a genetic diagnosis to each IEIs patient and represent the basis for the development of targeted therapies (58). However, a large fraction of patients with CVIDs may have complex genetics influenced by copy number and epigenetics modifications (58). Our results measuring the *in-vivo* response to vaccination start to sharpen the CVIDs landscape into more defined clinical diseases and defective immunologic mechanisms.

## Data availability statement

The datasets presented in this study can be found in online repositories. The names of the repository/repositories and accession number(s) can be found below: PRJNA967329 (SRA).

## Ethics statement

The studies involving human participants were reviewed and approved by The study was approved by the Ethical Committee of the Sapienza University of Rome (Prot. 0521/2020, July 13, 2020) and performed in accordance with the Good Clinical Practice guidelines, the International Conference on Harmonization guidelines, and the most recent version of the Declaration of Helsinki. The patients/participants provided their written informed consent to participate in this study.

## Author contributions

EP, VM, CAI, ST, AF, StDC, MG, MS, SF, VB and SA acquired the data. EP, FP, IQ, and RC were responsible for the analysis of the data and drafted the article. SeDC, EP, and RC performed the unsupervised analysis. CAg, CQ, FL GDN, DG, ES, CM, GG, AP, LB, SZ and CC critically revised the manuscript for important intellectual content. All authors approved the final version to be published and agree to be held accountable for all aspects of the work. RC, and FL made substantial contributions to the acquisition of funding. All authors contributed to the article and approved the submitted version.
