# Supplementary material for: Functional CVIDs phenotype clusters identified by the integration of immune parameters after BNT162b2 boosters
Source: Front Immunol. 2023 May 25;14:1194225. doi: 10.3389/fimmu.2023.1194225 (PMC10248522; doi:10.3389/fimmu.2023.1194225)
Supplement: Supplementary file 1 [file DataSheet_1.docx]

Supplementary Material

**Functional CVIDs phenotype clusters identified by the integration of immune parameters after BTN162b2 boosters**

Piano Mortari E.^1,2^ ^†^, Pulvirenti F.^3^ ^†^, Marcellini V.^4^, Terreri S.^1^, Fernandez Salinas A.^1^ Ferrari S.^5^, Di Napoli G.^2^, Guadagnolo D.^6^, Sculco E.^2^, Albano C.^1^, Guercio M.^7^, Di Cecca S.^7^, Milito C.^2^, Garzi G.^2^, Pesce A.M.^3^, Bonanni L.^3^, Sinibaldi M.^7^, Bordoni V.^7^, Di Cecilia S.^8^, Accordini S.^9^, Castilletti C.^9^, Agrati. C.^7^, Quintarelli C.^7^, Zaffina S.^10^, Locatelli F.^6,11^, Carsetti R.^1#^^, Quinti I.^2#^

^1^B Cell Unit, Immunology Research Area, Bambino Gesù Children's Hospital, IRCCS, Rome, Italy

^2^Department of Molecular Medicine, Sapienza University of Rome, Rome, Italy

^3^Reference Centre for Primary Immune Deficiencies, Azienda Ospedaliera Universitaria Policlinico Umberto I, Rome, Italy

^4^ Research Biobank, Bambino Gesù Children's Hospital, IRCCS, Rome, Italy

^5^Medical Genetics Unit, IRCCS Azienda Ospedaliero-Universitaria di Bologna, Bologna, Italy

^6^Department of Experimental Medicine, Policlinico Umberto I Hospital, Sapienza University of Rome, Rome, Italy.

^7^Department of Onco-Haematology, and Cell and Gene Therapy, Bambino Gesù Children’s Hospital, IRCCS, Rome, Italy

^8^FlowJo, BD Life-Sciences-Biosciences, Ashland OR, USA

^9^Department of Infectious, Tropical Diseases and Microbiology, IRCCS Sacro Cuore Don Calabria Hospital, Negrar di Valpolicella, Verona, Italy

^10^Occupational Medicine/Health Technology Assessment and Safety Research Unit, Clinical-Technological Innovations Research Area, Bambino Gesù Children’s Hospital, IRCCS, Rome, Italy

^11^Catholic University of the Sacred Heart, Rome, Italy

**†co-first, equally contribution**

**# co-last, equally contribution**

**Correspondence:**Dr. Rita Carsetti, B cell unit, Immunology Research Area, Bambino Gesù Children’s Hospital, IRCCS, 00146 Rome, Italy [rita.carsetti@opbg.net](mailto:rita.carsetti@opbg.net); ORCID: 0000-0002-2632-1078

# Supplementary Figures and Tables

**
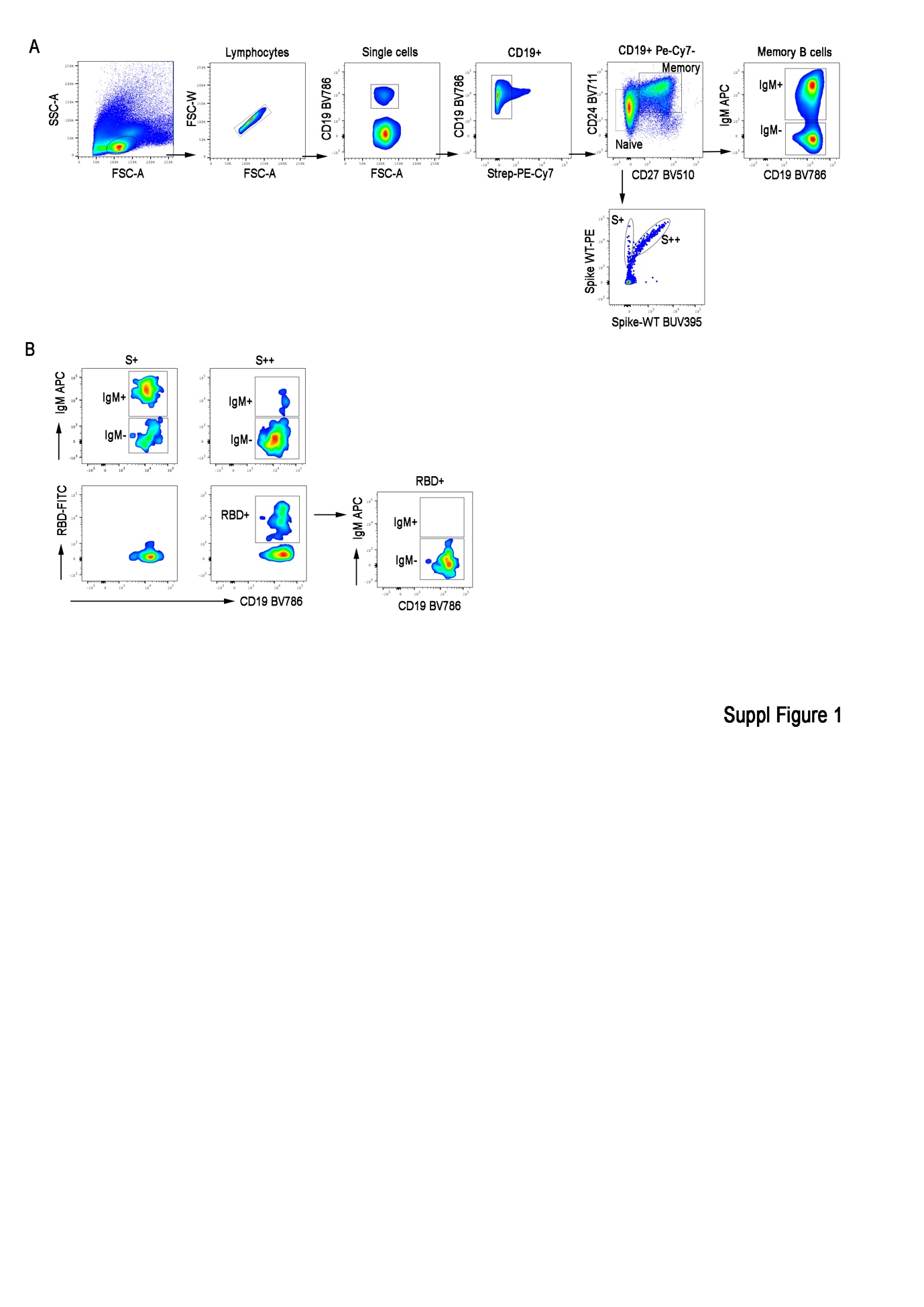
**

**
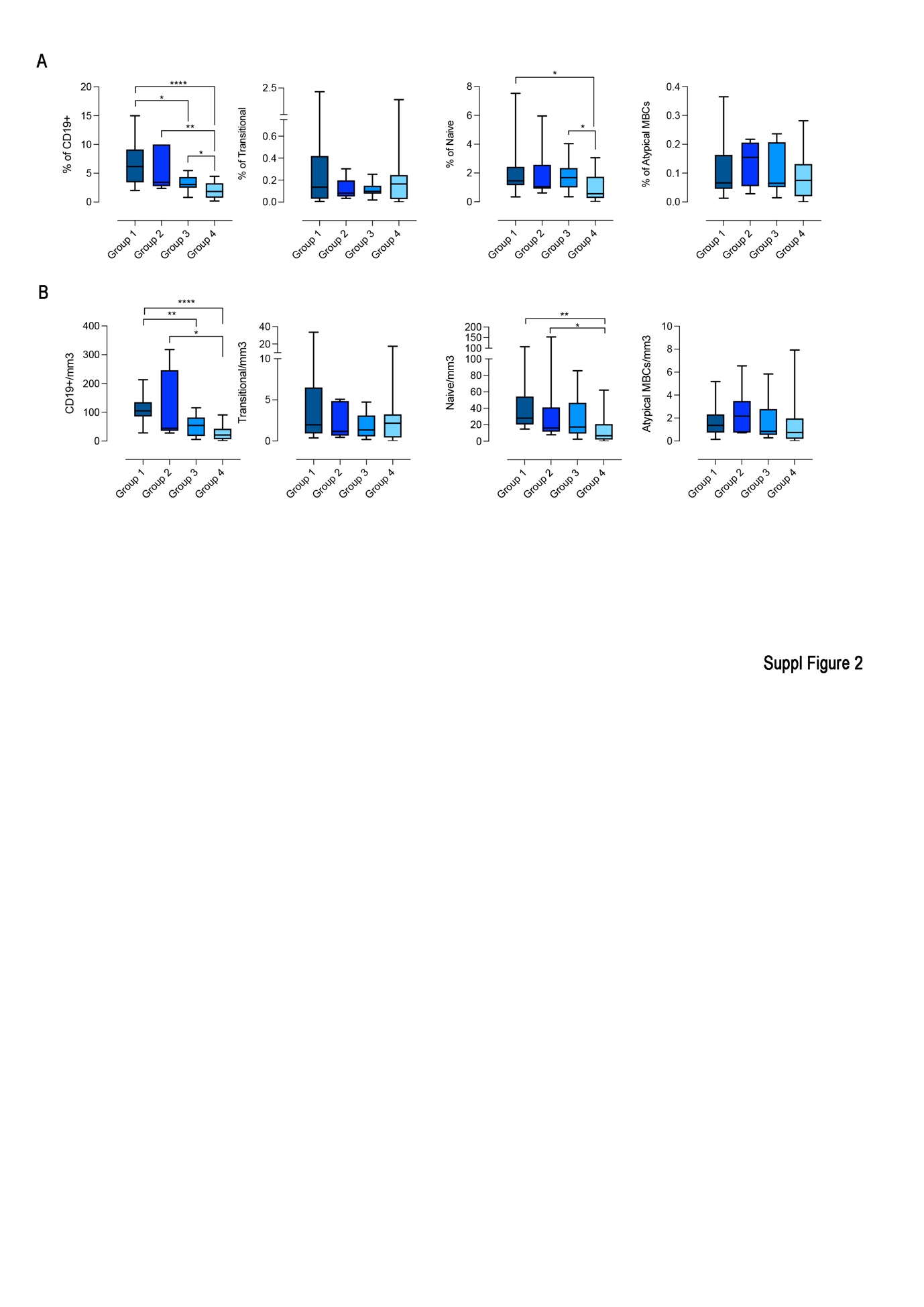
**

**
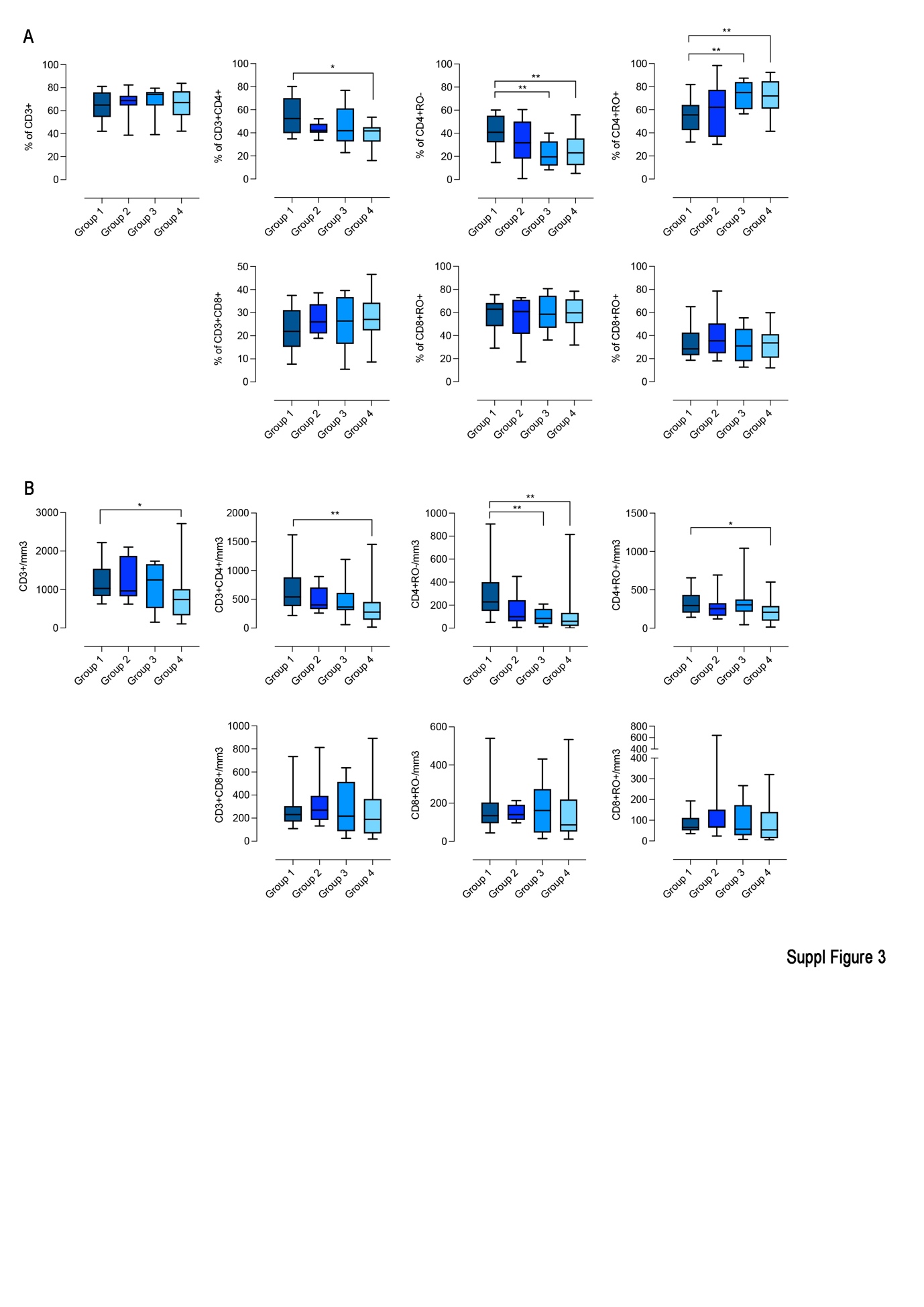
**

**
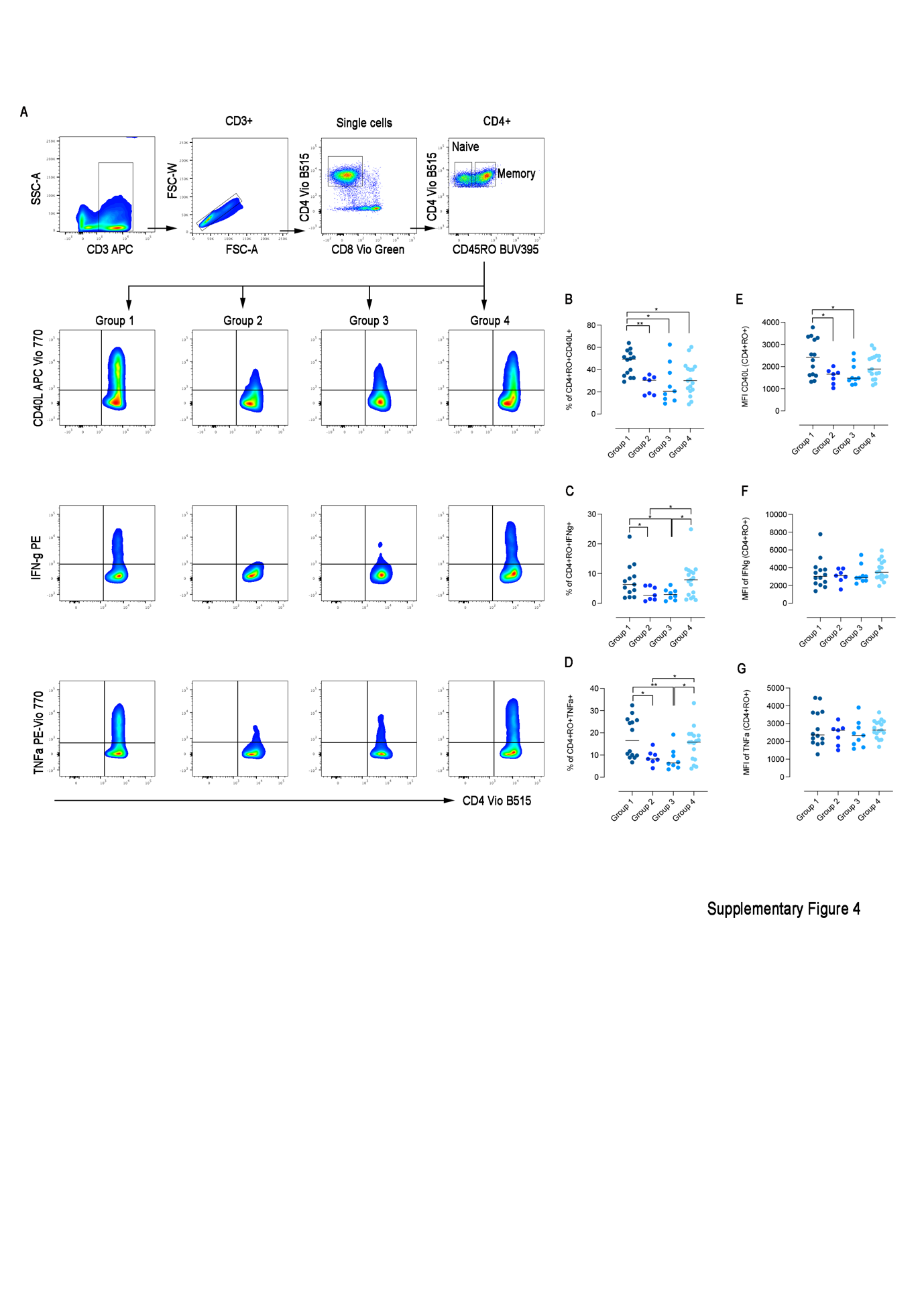
**


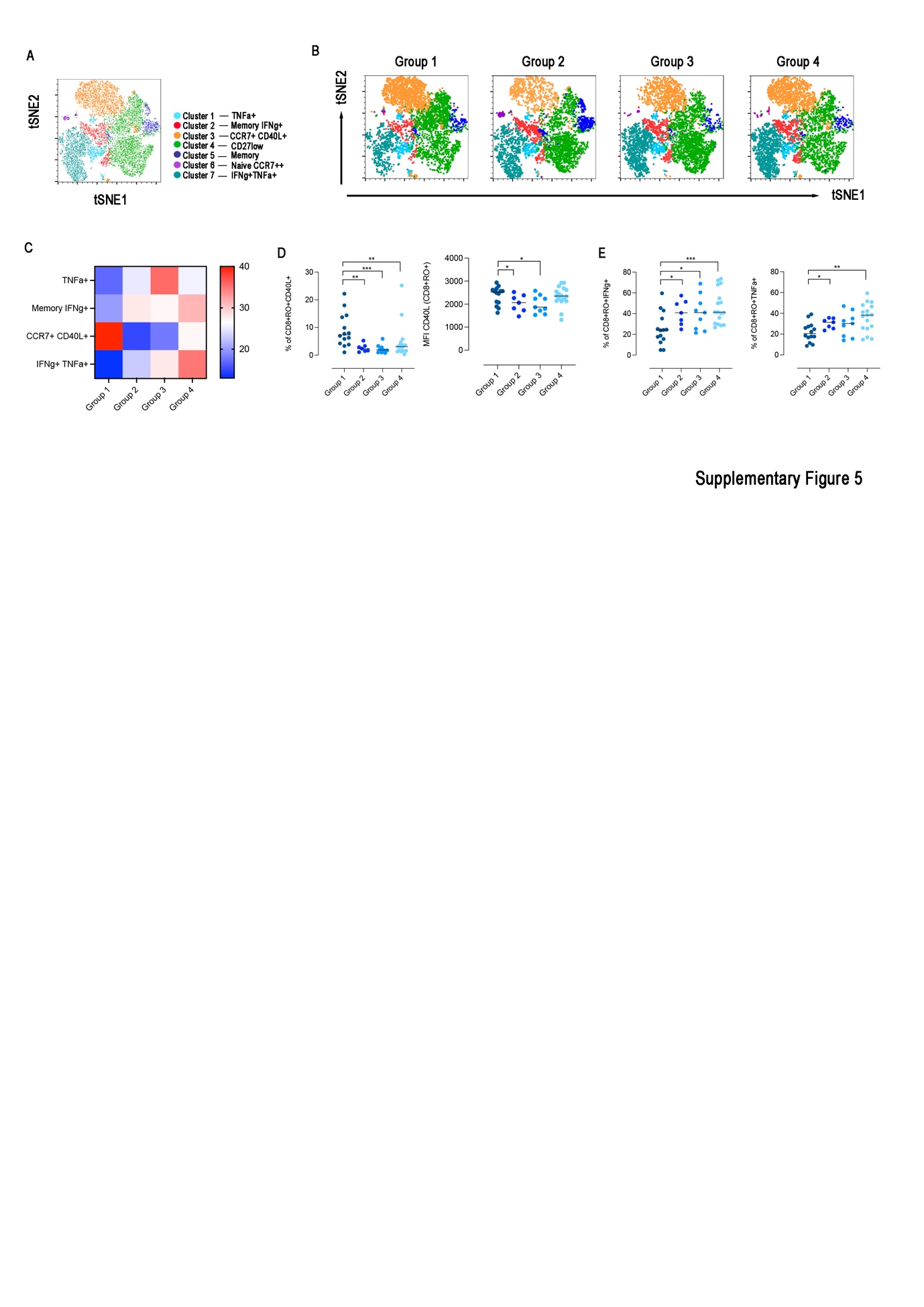


**Supplementary Figure 1. Gating strategy.** (A) FACS plots depict the gating strategy for the identification of total (CD19+CD24+CD27+), IgM+ and IgM- MBCs, in a representative HCW. Low (S+) and high (S++) affinity spike-specific MBCs are shown. (B) IgM+, IgM- and RBD+ MBCs among S+ and S++. IgM expression on RBD+ MBCs is shown.

**Supplementary Figure 2. Frequency and absolute B-cell number in the 4 groups***.* (A-B) Bar plots depict the frequency (A) and the absolute numbers (B) of total B cells (CD19+), transitional B cells (CD19+CD24++CD38++), mature-naive B cells (CD19+CD24+CD27-) and atypical MBCs (CD19+CD24-CD27-CD38-) in the four groups of CVIDs patients. The frequency of the B cell populations was evaluated in the lymphocyte gate. Non-parametric Mann–Whitney t-test was used to evaluate statistical significance. Two-tailed P value significances are shown as * p<0.05, **p< 0.01, ***p< 0.001, ****p< 0.0001.

**Supplementary Figure 3. Frequency and absolute T-cell number in the 4 groups***.* (A-B) Bar plots depict the frequency (A) and the absolute number (B) of total T cells (CD3+), CD4+ (CD3+CD4+), and CD8+ T cells (CD3+CD8+), naïve CD4+ (CD3+CD4+RO-) and CD8+ (CD3+CD8+RO-), and memory CD4+ (CD3+CD4+RO+) and CD8+ (CD3+CD8+RO+). Non-parametric Mann–Whitney t-test was used to evaluate statistical significance. Two-tailed P value significances are shown as * p<0.05, **p < 0.01.

**Supplementary Figure 4. CD40L and cytokines secreting memory CD4+ cells.** (A) FACS plots show the gating strategy used to identify CD4+ memory T cells (CD3+CD4+CD45RO+) positive for CD40L, IFNγ and TNFα (intracellular staining) after stimulation with CytoStim in one patient representative of each group. Frequency of CD4+ memory T cells expressing CD40L (B), IFNγ (C) and TNFα (D) in the different groups. Mean Fluorescence Intensity (MFI) of CD40L (E), IFNγ (F) and TNFα (G) (gated on CD4+ memory T cells). All patients were analyzed after the 3^rd^ dose. Bars indicate the median. Non-parametric Mann–Whitney t-test was used to evaluate statistical significance. Two-tailed P value significances are shown as * p<0.05, **p < 0.01.

**Supplementary Figure 5. CD8+ T-cell population in the 4 groups.** (A) X-Shift B-cell cluster sets originated from the four concatenated groups and overlaid onto the Opt-SNE map, each cluster is indicated by a color. (B) Merged Opt-SNE plots for each group with relative X-Shift cluster sets overlaid onto the Opt-SNE map. (C) Heat map depicts the abundancy (%) of cluster 1, 2, 3 and 7 in the four groups. Frequency of CD8+ memory T cells expressing CD40L (D), (E) IFNγ and TNFα in the different groups. All patients were analyzed after the 3^rd^ dose.

Bars indicate the median. Non-parametric Mann–Whitney t-test was used to evaluate statistical significance. Two-tailed P value significances are shown as * p<0.05, **p < 0.01, ***p<0.001.

**Supplementary Table 1**. List of 53 genes included in the IEIs panel.

| **Gene** | | | | | | |
| --- | --- | --- | --- | --- | --- | --- |
| ADA | CD19 | CR2 | IL2RA | NFKBIA | RAG1 | TNFRSF13C |
| AICDA | CD3G | CTLA4 | KRAS | NRAS | RAG2 | TTC7A |
| ATM | CD40 | DKC1 | LIG1 | OAS1 | SH2D1A | UNG |
| BLNK | CD40L | DCLRE1 | LRBA | PIK2CD | STAT1 | WAS |
| BTK | CD79A | FAS | MAGT1 | PIK3R1 | STAT3 | XIAP |
| CASP8 | CD79B | FASL | MS4A1 | PLCG2 | STX11 |  |
| CASP10 | CD81 | ICOS | NFKB1 | PRF1 | TCF3 |  |
| CD179B | CECR1 | IKZF1 | NFKB2 | PRKCD | TNFRSF13B |  |

**Supplementary Table 2**. Anti-S1 and neutralizing antibodies levels and frequencies of peripheral specific B-cells measure of d at the different study time in CVID patients naïve to SARS-CoV-2 infection.

|  | **Group 1(S1 IgG+/S++ MBC+)** | | | **Group 2 (S1 IgG+/S+ MBC+)** | | | **Group 3 (S1 IgG+/S+ MBC)** | | | **Group 4 (S1 IgG-/S+ MBC-)** | | |
| --- | --- | --- | --- | --- | --- | --- | --- | --- | --- | --- | --- | --- |
|  | **n=14** | | | **n=7** | | | **n=9** | | | **n= 17** | | |
|  | Median | IQR | | Median | IQR | | Median | IQR | | Median | IQR | |
| S1 IgG post 3^rd^ (BAU/ml) | 967.8 | 616.9 | 2394 | 178.5 | 20.85 | 617.6 | 99.44 | 51.85 | 540.4 | 2.95 | 1.03 | 6.46 |
| S1 IgG pre 4^th^ (BAU/ml) | 139.4 | 98.92 | 341.4 | 90.96 | 20.31 | 140.5 | 50.69 | 23.74 | 164.9 | 11.89 | 3.79 | 55.37 |
| S1 IgG post 4^th^ (BAU/ml) | 593.0 | 205.5 | 2520 | 140.5 | 65.88 | 570.9 | 82.36 | 40.61 | 136.6 | 5.3 | 2.67 | 10.69 |
| MNA90 (WT) reciprocal of dilution post 3^rd^ | 120 | 40 | 160 | 20 | >10 | 40 | 10 | >10 | 65 | >10 | >10 | >10 |
| MNA90 (WT) reciprocal of dilution pre 4^th^ | 20 | 10 | 40 | 7.5 | >10 | 20 | >10 | >10 | 17.5 | >10 | >10 | >10 |
| MNA90 (WT) reciprocal of dilution post 4^th^ | 160 | 70 | 200 | 30 | >10 | 50 | 7.5 | >10 | 17.5 | >10 | >10 | >10 |
| MNA90 (BA.5) reciprocal of dilution post 3^rd^ | >10 | >10 | >10 | >10 | >10 | >10 | >10 | >10 | 5 | >10 | >10 | >10 |
| MNA90 (BA.5) reciprocal of dilution pre 4^th^ | >10 | >10 | >10 | >10 | >10 | >10 | >10 | >10 | 17.5 | >10 | >10 | >10 |
| MNA90 (BA.5) reciprocal of dilution post 4^th^ | >10 | >10 | 12.5 | >10 | >10 | >10 | >10 | >10 | 5 | >10 | >10 | >10 |
| S+ MBC post 3^rd^ (%) | 0.26 | 0.22 | 0.33 | 0.13 | 0.12 | 0.22 | 0 | 0 | 0 | 0 | 0 | 0 |
| S+ MBC pre 4^th^ (%) | 0.47 | 0.37 | 0.59 | 0.37 | 0.14 | 0.68 | 0 | 0 | 0 | 0 | 0 | 0 |
| S+MBC post 4^th^ (%) | 0.50 | 0.39 | 0.79 | 0.33 | 0.18 | 0.67 | 0 | 0 | 0.37 | 0 | 0 | 0 |
| S++ MBC post 3^rd^ (%) | 0.09 | 0.06 | 0.39 | 0 | 0 | 0 | 0 | 0 | 0 | 0 | 0 | 0 |
| S++ MBC pre 4^th^ (%) | 0.11 | 0.06 | 0.37 | 0 | 0 | 0 | 0 | 0 | 0 | 0 | 0 | 0 |
| S++ MBC post 4^th^ (%) | 0.44 | 0.22 | 0.55 | 0.02 | 0 | 0.13 | 0 | 0 | 0 | 0 | 0 | 0 |
| RBD+ post 3^rd^ (%) | 10.1 | 7.4 | 19.5 | 0 | 0 | 0 | 0 | 0 | 0 | 0 | 0 | 0 |
| RBD+ pre 4^th^ (%) | 15.8 | 4.7 | 24.6 | 0 | 0 | 1.5 | 0 | 0 | 0 | 0 | 0 | 0 |
| RBD+ post 4^th^ (%) | 22.9 | 13.3 | 32.9 | 8.9 | 0 | 19.5 | 0 | 0 | 0 | 0 | 0 | 0 |

**Supplementary Table 3. Polymorphism analysis**

| **GROUP** | **SEX** | **AGE** | **GENE** | **VARIANT cDNA** | **VARIANT protein** | **Interpretation** | **ZIGOSITY** | **allele frequency**  **(Gnomad v2.1.1 Exomes)** | **Associated OMIM phenotype (constitutional variants only)** | **patient phenotype** |
| --- | --- | --- | --- | --- | --- | --- | --- | --- | --- | --- |
| 1 | F | 72 | TNFRSF13B (NM_012452.3) | c.515G>A | p.Cys172Tyr | VUS (PM2 moderate, PP3 supporting) | het | 0.0183% | CVID 2 (AD/AR) | infection only |
|  |  |  | LRBA (NM_001364905.1) | c.6433C>T | p.Arg2145Cys | VUS (PM2 moderate, PP3 supporting) | het | 0.0012% | CVID 8 with autoimmunity (AR) |  |
| 3 | M | 38 | DCLRE1C (NM_001033855.3) | c.1561C>A | p.Leu521Ile | VUS (PM2 moderate, BP4 supporting) | het | 0.0008% | Omenn syndrome 603554 AR; SCID, Athabascan type (AR) | Autoimmunity (ICP), GLILD, INCPH |
|  |  |  | IGLL1 (NM_020070.4) | c.301G>A | p.Gly119Arg | VUS (PM2 moderate, BP4 supporting) | het | 0.0016% | Agammaglobulinemia 2 (AR) |  |
| 3 | M | 38 | PLCG2 (NM_002661.5) | c.3368A>C | p.Glu1123Ala | VUS (PM2 moderate) | het | n/a | Autoinflammation, antibody deficiency, and immune dysregulation syndrome (AD); Familial cold autoinflammatory syndrome 3 (AD) | GLILD |
| 4 | F | 62 | FAS (NM_000043.6) | c.580G>A | p.Glu194Lys | LB (PP2 supporting, BS1 strong, BS2 supporting) | het | 0.1544% | ALPS, IA (AD) | Autoimmunity (alopecia) |
| 4 | F | 65 | STX11 (NM_003764.4) | C.616G>A | P.Glu206Lys | VUS (BS1 strong) | het | 0.1067% | Hemophagocytic lymphohistiocytosis 4 (AR) | Infection only |

**
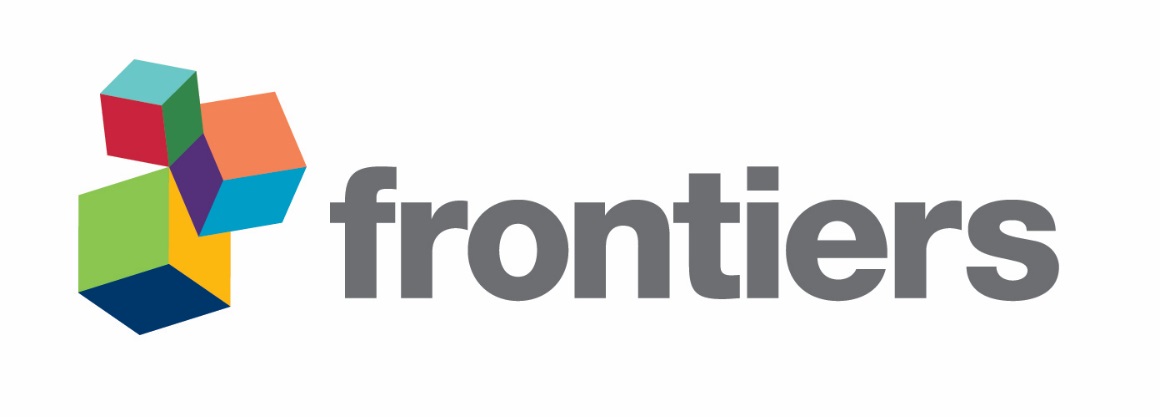
**
